# Limited vs. Extended Resection of Stanford Type A Acute Aortic Dissections

**DOI:** 10.3390/medicina60081245

**Published:** 2024-07-31

**Authors:** Suvitesh Luthra, Pietro G. Malvindi, Miguel M. Leiva-Juárez, Hannah Masraf, Davorin Sef, Szabolcs Miskolczi, Theodore Velissaris

**Affiliations:** 1 Division of Cardiac Surgery, Wessex Cardiothoracic Centre University Hospital Southampton, Southampton SO16 6YD, UK; 2 Academic Unit of Human Development and Health, Faculty of Medicine, University of Southampton, Southampton SO16 6YD, UK; 3 Cardiac Surgery Unit, Lancisi Cardiovascular Center, Ospedali Riuniti delle Marche, Polytechnic University of Marche, 60121 Ancona, Italy; 4 Department of Cardiothoracic Surgery, Beth Israel Deaconess Medical Center, Boston, MA 02215, USA; 5 Kingston Hospital NHS Foundation Trust, Kingston upon Thames KT2 7QB, UK; 6 Department of Cardiac Surgery, University Hospital Leicester NHS Trust, Leicester LE5 4PW, UK

**Keywords:** acute aortic dissection, type A, Stanford type A

## Abstract

*Background and Objectives:* This is a propensity-matched, single-center study of limited versus extended resection for type A acute aortic dissection (AAAD). *Materials and Methods*: This study collected retrospective data for 440 patients with acute type A aortic dissection repairs (limited resection, LR-215; extended resection, ER-225), of which 109 pairs were propensity-matched to LR versus ER. Multivariate analysis was performed for inpatient death, long-term survival and the composite outcome of inpatient death/TIA/stroke. Kaplan–Meier survival curves were compared at 1, 3, 5, 10 and 15 years using the log-rank test. *Results*: Mean age was 66.9 ± 13 years and mean follow-up was 5.3 ± 4.7 years. A total of 48.9% had LR. In-hospital mortality was 10% (LR: 6% vs. ER: 13.8%, *p* < 0.01). ER, NYHA class, salvage surgery and additional procedures were predictors of increased mortality in unmatched data. Propensity-matched data showed no difference in TIA/stroke rates, LOS, inpatient mortality or composite outcomes. LR had better survival (LR: 77.1% vs. ER: 51.4%, *p* < 0.001). ER (OR: 1.97, 95% CI: 1.27, 3.08, *p* = 0.003) was a significant predictor of worse long-term survival. At 15 years, aortic re-operation was 17% and freedom from re-operation and death was 42%. *Conclusions*: Type A aortic dissection repair has high mortality and morbidity, although results have improved over two decades. ER was a predictor of worse perioperative results and long-term survival.

## 1. Introduction

Stanford type A acute aortic dissection (AAAD) represents a life-threatening surgical emergency. Surgical mortality remains high despite several advances in operative techniques, neuro-cerebral monitoring, neuroprotection and postoperative care [1,2].

Clinical presentation and the extent of surgery remain the major determinants of early and late outcomes, in addition to operator experience and surgical volumes. Data on medium- and long-term survival, however, remain scarce, even in large international databases. There remain several controversies regarding cannulation strategies, neuroprotection, the identification and resection of tears, closed versus open distal anastomosis, the extent of resection and the fate of residual ‘downstream’ aorta and its impact on late re-interventions and survival.

Recently, there has been a further proliferation of devices that enable a single-stage stabilization of the downstream aorta with an extended resection with an open hybrid stent graft at the time of the primary repair of AAAD. This also facilitates the resection and/or stabilization of difficult tears, especially in the arch and proximal descending thoracic aorta. Extended resections with resection of the arch and reattachment of the head and neck vessels are technically much more challenging than limited resections of the ascending aorta. The role and indications for extended resections for AAAD remain undefined. Guidelines suggest that they should be undertaken by aortic surgeons in high volume aortic centers only.

Extended resections are associated with longer cross-clamp times, bypass times and operative times. Periods of deep hypothermic circulatory arrest and cerebral perfusion also contribute to the increased morbidity of adverse neurological outcomes. The long-term neurological sequelae of extended resections, including those on memory and motor function, are not well known.

Our working hypothesis based on conventional wisdom was that extended resections have greater morbidity and mortality in the perioperative period and worse long-term survival compared to limited resections.

The aims of this study were as follows:

1. To evaluate the perioperative outcomes and late survival of AAAD repair;
2. To compare limited versus extended resections.

## 2. Materials and Methods

### 2.1. Population

This study was conducted as per the STROBE (Strengthening the reporting of observational studies in epidemiology) guidelines. Data on AAAD repairs were retrospectively collected (January 2000–January 2019) from the PAS (Patient Administration System). AAAD was defined as any dissection that involved the ascending aorta and presented within 2 weeks of symptoms. Patients who refused the operation, underwent conservative management or died before the operation were excluded. Data were processed in compliance with institutional data policies (SEV/0697). Consent for individual use was waived due to the nature of this study and prior approval at the time of consent. Long-term survival statistics were collected from the Patient Administration System (e-CAMIS) and the National Healthcare Service Spine Portal Summary Care Records (SCR). Baseline demographics included age, sex, New York Heart Association (NYHA) class, Canadian Cardiovascular Society (CCS) class, prior cardiac surgery, diabetes, hypertension, smoking status, renal and pulmonary disease, obesity (BMI > 28), peripheral vascular disease, left ventricular ejection fraction, emergent or salvage surgery, aortic arch procedure, additional cardiac procedure, unstable status and EuroSCORE. Emergency surgery was defined as that performed before the next day after the decision for surgery was made. Salvage was defined as requiring cardiopulmonary resuscitation en route to the theater or prior to the induction of anesthesia.

### 2.2. Operative Techniques

All operations were performed through a median sternotomy. The cannulation site was chosen according to aortic CT findings, technical feasibility of access and surgeon’s preferred techniques [3]. Cold-blood cardioplegia was used for myocardial protection. The ascending aorta was replaced with an interposition graft. The extent of aortic resection (ascending aorta, hemi-arch or arch with conventional or frozen elephant trunk operation) and aortic root repair or replacement and any other additional procedures were based on preoperative scans, trans-esophageal echocardiogram, intraoperative inspection, the location of tears and surgeon’s preference.

Limited resections (LRs) included ascending aorta resection and interposition grafts with a closed distal anastomosis. Extended resections (ERs) included resections of the distal ascending aorta, hemi-arch or arch with/without the re-implantation of neck vessels or variations of the frozen elephant trunk operation. An open distal aortic reconstruction or frozen elephant trunk operation under deep hypothermic circulatory arrest (DHCA) was usually performed in the presence of an intimal tear extending into the aortic arch or crossing the clamp area. Cerebral protection for open anastomosis was achieved with either DHCA or DHCA with antegrade/retrograde cerebral perfusion. Near-infrared spectroscopy was used after 2010 for noninvasive, continuous cerebral oxygen saturation monitoring of balance between oxygen delivery and extraction of the cerebral cortex. Infrared light-emitting diode sensors were bilaterally placed on the forehead for measurement. Bilateral similar values above 60% were maintained. These correlate with venous cerebral saturations. Higher perfusion pressures or selective perfusion with cannulation of the left carotid ostium from inside the arch was used for persisting low levels.

### 2.3. Statistical and Propensity Score Analysis

Distributions were visually inspected and normality was tested by a Shapiro-Wilcoxon W test. Continuous variables were compared using a Mann–Whitney test (or Student’s *t*-test for normal distributions) and categorical variables by Fisher’s exact test or chi-squared test. Sixteen preoperative variables were identified. Variables with *p* < 0.2 on univariate analysis were entered into a multivariate analysis to identify independent predictors of inpatient death and the composite outcome of inpatient death/TIA/stroke. The arbitrary *p* < 0.2 value provides enough predictive value even without statistical significance to warrant further investigation in the multivariate analysis. A more stringent lower value would make the model too exacting at risk of excluding clinically relevant variables that are known to affect outcomes from experience and from a review of the literature. The clinically relevant non-intercorrelated variables that have been known to predict adverse perioperative outcomes and worse survival in the literature were included in the multivariable analysis to test their predictive value in addition to statistical significance.

Kaplan–Meier survival curves were compared at 1, 3, 5, 10 and 15 years using the log-rank test. The Cox proportional hazards model was used to determine predictors of long-term survival. The proportionality assumption was tested with Schoenfeld residuals. Propensity scores were calculated using logistic regression and ER patients were posteriorly matched with LR patients at a 1:1 ratio using the nearest neighbor method, with a caliper width of 0.2 of the standard deviation of propensity score logit. Means, standard deviations and proportions of baseline demographics were compared to assure appropriate balance; statistical significance was tested using a Wilcoxon signed-rank paired test and McNemar’s test for continuous and categorical variables, respectively. Standardized differences were used for appropriate balance for bias < 10% (Supplementary Figure S1).

## 3. Results

AAAD repair was performed in 440 patients [LR—215 (48.7%), ER—225 (51.3%)] (Supplementary Table S1). Mean age was 66.9 ± 13 years and mean follow-up was 5.3 ± 4.7 years. A total of 48.9% of the patients had LR. A total of 16.8% were unstable at presentation and 8% needed a salvage operation; 20% had an additional cardiac procedure. A fifth of the patients had an arch procedure (hemi-arch, total arch or a frozen elephant trunk operation). One hundred nine patients with ER were propensity-matched to patients with LR (Supplementary Table S2).

### 3.1. Operative Results

There was no significant difference in bypass times (LR: 173 min vs. ER: 191 min, *p* = 0.225) and cross-clamp times (LR: 99 min vs. ER: 99 min, *p* = 0.862) (Table 1). Mean total circulatory arrest time for ER was 27 min (IQR: 18–33.5 min). Overall, the TIA/stroke rate was 18.4%. There was no difference in perioperative TIA/stroke (LR: 16.3% vs. ER: 20.4%, *p* = 0.474). Overall length of stay was 12 days (IQR—8–20 days, LR: 12 days vs. ER: 11 days, *p* = 0.012). In-hospital mortality was 10% (LR: 6% vs. ER: 13.8%, *p* < 0.01).

After propensity matching, there was no difference between LR and ER in TIA/stroke rates, length of stay, inpatient mortality or composite outcomes (Table 1).

ER (OR: 2.92, 95% CI: 1.36, 6.29, *p* = 0.006), NYHA class (OR: 2.49, 95% CI: 1.10, 5.63, *p* = 0.029), salvage surgery (OR: 3.60, 95% CI: 1.27, 10.17, *p* = 0.016) and additional cardiac procedures (OR: 2.23, 95% CI: 1.01, 4.92, *p* = 0.047) were predictors of increased inpatient mortality (Supplementary Table S3). Salvage surgery, bypass time and age > 70 yrs were predictors of worse composite outcomes (Table 2). Operative mortality for salvage and non-salvage cases was 25.7% vs. 8.6% (*p* = 0.001).

ER was a predictor of increased inpatient mortality (OR: 2.92, CI: 1.36, 6.29, *p* = 0.006), worse composite outcomes (OR: 1.44, CI: 0.91, 2.29, *p* = 0.12) and reduced long term survival (OR: 1.97, CI: 1.27, 3.08, *p* = 0.003).

Cox regression identified ER (OR: 1.97, 95% CI: 1.27, 3.08, *p* = 0.003), NYHA class (OR: 2.08, 95% CI: 1.30 to 3.33, *p* = 0.002), prior cardiac surgery (OR:1.75, 95% CI: 1.02 to 2.99, *p* = 0.041) and age > 70 years (OR: 2.00, 95% CI: 1.34 to 3.00, *p* = 0.001) as significant predictors of worse long term survival (Table 3).

**Figure 1:**
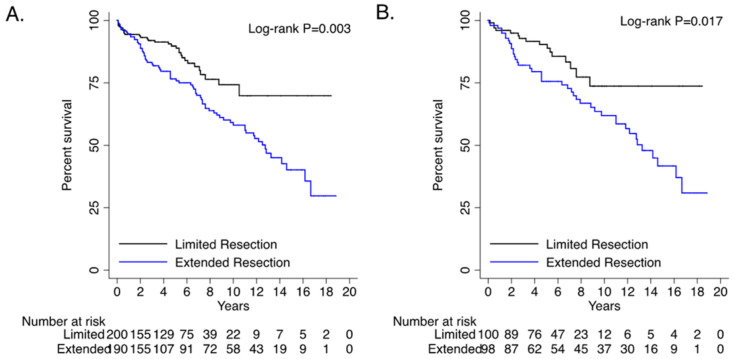
Survival by extent of resection (limited versus extended). (**A**) Unmatched; (**B**) matched.

Survival in propensity-matched pairs was better for LR (LR: 77.1% vs. ER: 51.4%, *p* < 0.001) (Table 3, Figure 1). Patients with DHCA and cerebral perfusion had worse long-term survival (Supplementary Figure S2). Age > 75 did not have worse survival (Supplementary Figure S3).

**Figure 2:**
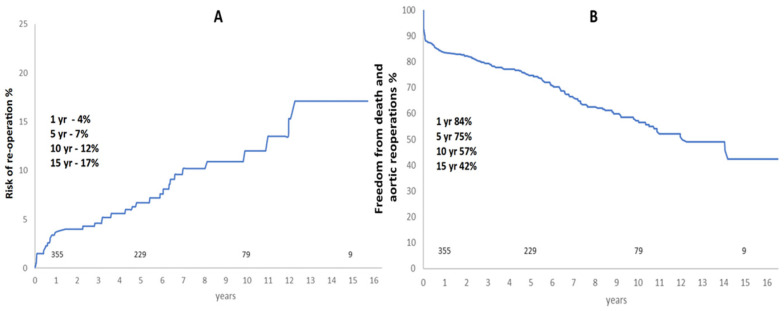
(**A**)—Risk of re-operation (%) on any part of the aorta after acute aortic dissection repair. (**B**)—Freedom from composite outcome of death and aortic re-operation (%) after acute aortic dissection repair.

Incidence of aortic re-operation was 17% at 15 years and freedom from re-operation and death was 42% at 15 years (Figure 2). Most late re-operations were aortic valve- or root-related (Table 4).

### 3.2. Comment

Institutional studies and registries have reported a mortality of 17–28% for AAAD repair (GERAADA (2006-14): 19.5%, JCSD (2008-15): 9.5%, IRAD (2010-13): 18.4%, NORCAAD (2005-14): 17.6%) [4,5,6,7]. The overall mortality in our series was 10% (2000-19) with changes in operative techniques, use of ante-flow cannulation, antegrade cerebral perfusion and use of near-infrared spectroscopy. Stroke remains a devastating complication. STS NACSD (2014-17) reported a stroke rate of 13% [8]. Axillary cannulation was associated with a lower risk of stroke versus femoral (odds ratio, 0.60; *p* < 0.001). Retrograde cerebral perfusion (odds ratio, 0.75; *p* = 0.008) was associated with a reduced risk compared with no cerebral perfusion or antegrade cerebral perfusion. Total arch replacement, longer circulatory arrest time, cerebral perfusion time and cardiopulmonary bypass time were all related to a higher risk of postoperative stroke. Critical status, preoperative hypotension, shock or cardiopulmonary resuscitation, malperfusion syndrome and neurologic deficit were associated with poor operative outcomes.

Age > 70 years was an independent risk factor for mortality in the IRAD registry. Surgery was significantly better than medical management even in patients > 80 yrs. In the JCSD registry (mean age 69 yrs), age was a significant risk factor for both limited and extended resections involving the arch. GERAADA showed a significantly higher in-hospital mortality for octogenarians (OR: 3.23, 34.9% vs. 15.8%, *p* < 0.001). In our experience, age > 70 years was not a predictor of inpatient death but adversely affected the composite outcome of TIA/stroke/inpatient death (OR: 1.69, 95% CI: 1.05, 2.71, *p* = 0.031). Age > 70 years was also a strong predictor of poor late survival.

Our results are striking for the following reasons:

(a) Propensity-matched in-hospital mortality and composite outcomes were similar for ER and LR.
(b) Long-term survival for extended resections was worse than for limited resections.
(c) The risk of aortic re-operation was very low, at 10% at 10 years and 17% at 15 years.

Evidence for the extent of resection remains conflicting and results differ for specialist and non-specialist units. Between 2004 and 2016, the STS Adult Cardiac Surgery Database reported that AAAD repairs involved the ascending aorta only in 54% of patients and arch interventions in 46% [9]. Early mortality remained high regardless of the extent of aortic resection (30-day mortality was 18.9% for ascending only operations vs. 19.8% for arch resections, *p* = 0.09). However, the Japanese experience with more extensive resections and arch intervention in AAAD repair, especially with frozen elephant trunk (FET) operations, has shown consistently better results. In 426 AAAD repairs, Asakura et al. had no difference in thirty-day mortality and neurological dysfunction between the FET and non-FET groups (1.4% vs. 2.4%, *p* = 0.50 and 5.0% vs. 6.3%, *p* = 0.61, respectively) [10]. Long-term survival was better with FET (*p* = 0.008). Freedom from distal thoracic re-intervention was similar (*p* = 0.74). After propensity matching, freedom from aortic-related death was better with FET (*p* = 0.044). In the ARCH database, 978 patients who underwent total aortic arch replacement for AAAD with or without FET showed significant differences in terms of permanent neurologic deficits (11.9% vs. 10.1%, *p* = 0.59) and spinal cord injury (4.0% vs. 6.3%, *p* = 0.52). After post hoc propensity score stratification, FET was associated with a significantly lower mortality risk (odds ratio, 0.47; *p* = 0.03). In a pooled meta-analysis of 38 studies by Smith et al., hospital mortality for extended arch techniques was 8.6% (95% CI 7.2–10.0) (11.9% for total arch, 8.6% total arch and frozen stented elephant trunk, 6.3% hemi-arch and frozen stented elephant trunk and 5.5% total arch and ‘warm’ stent graft) [11]. The rate of stroke was 5.7% (95% CI 3.6–8.2) and that of spinal cord ischemia was 2.0% (95% CI 1.2–3.0). There is an emerging expert consensus for the safety of concomitant repair of the downstream aorta in specialist aortic centers [12]. Extended resections have been shown to be safe, with excellent mid- and long-term outcomes of ‘downstream aortic re-modelling’ with various modifications of FET in AAAD repair at specialist aortic units.

Guidelines and consensus papers favor open distal repair in AAAD repair [13]. Despite the technical advantages in constructing a distal anastomosis with an excellent visualization while providing a more radical resection of the pathological wall, there is no evidence that this technique carries lower mortality or morbidity. A total of 50% of dissection repairs in this series were performed with a closed distal technique, compared to 14–41% in the literature. The choice between these two approaches for distal aortic repair was based mainly on anatomical factors associated with the dissection (i.e., extension beyond the ascending aorta, primary intimal tear located in the aortic root or the ascending aorta and need for arch intervention) but was influenced mainly by surgeon preferences and idiosyncrasies of individual techniques. Limited resections have the advantages of a quicker operation and avoiding prolonged hypothermic circulatory arrest and its attendant complications [14]. The disadvantages include cross-clamping the dissected aorta, with a risk of rupture and malperfusion, the inability to evaluate for additional tears, limited resection of the ascending aorta and more technically difficult distal anastomosis. AAAD dissection repair is essentially a lifesaving procedure that should be performed without increasing the complexity of the operation. Proponents of conservative resection argue that most emergency AAADs are referred to peripheral non-aortic referral centers, where the treating surgeons have limited proficiency and experience in complex emergency resections [15]. The need for re-interventions for ‘downstream problems’ of re-entry tears and growth of the distal aorta is less than 10–15% in a safe, elective setting with low mortality. This ‘less is more’ approach of limited ascending aortic resection is primarily aimed at preventing the life-threatening detumescence and rupture of the dissected aorta. A meta-analysis of nine studies found lower early mortality (RR = 0.69, *p* = 0.005) but a higher incidence of postoperative aortic events, including the re-operation of the distal aorta (RR = 3.14, *p* < 0.001), with limited proximal aortic resections [16]. The extent of aortic resection did not adversely affect perioperative stroke rates (RR: 0.73, 95% CI: 0.30–1.78, *p* = 0.50) or long-term survival (HR = 1.02, 95% CI 0.51–2.06, *p* = 0.96).

Is it necessary to identify and resect additional tears and does the false lumen patency affect long term survival? Kim et al. identified re-entry tears in the proximal descending thoracic aorta (38.5%), distal DTA (25.2%) and abdominal aorta (41.7%) in 309 non-syndromic AAAD repairs [17]. Multivariate analysis showed proximal DTA re-entry tears were an independent risk factor for aortic re-intervention (HR: 4.955; *p* < 0.01) and significant aortic expansion (HR: 4.214; *p* = 0.002). Lin et al. noted that a persistent false lumen presents a faster expansion of aortic diameter (B = 5.935, 95% CI: 0.35, 11.52; *p* = 0.038) and growth rate > 5 mm/year (*p* = 0.029) [18]. However, persistent false lumen did not predict post-discharge mortality (*p* = 0.479). Multiple other studies have shown that the number of distal tears adversely affects the rate of aortic growth, and false lumen patency is associated with late events and adverse long-term survival [19,20]. Open distal anastomosis is associated with higher rates of distal false lumen thrombosis. However, despite this presumed better evolution of the downstream aorta, the survival benefit of open distal anastomosis is not superior to that of closed distal anastomosis.

Long-term survival data are one of the strengths of our study. This compares favorably with other large single-institution studies. Mid- and long-term survival data are still scarce even in multicenter studies and registries. GERAADA has not yet provided mid-term data, while IRAD investigators only reported a 3-year survival rate for a minority of the patients enrolled in the registry.

In our experience, surgical re-interventions for the ‘downstream aorta’ were rare. Despite indications for surgery, some of these patients may have been managed conservatively, especially during the earlier periods of this study. The single most important determining factor for the extent of resection and survival is the surgeon. These arguments for lesser resection probably do not apply to high-volume, specialist aortic centers.

## 4. Limitations

This was a single-center, retrospective analysis. The vicissitudes and idiosyncrasies of operative techniques, with multiple surgeons in this series, spanning a period of two decades, remain an uncorrected confounder. There were significant improvements in operative monitoring and perioperative care in the second decade. These are historical data over two decades before the advances of covered and noncovered hybrid open stents. Information regarding the cause of death during follow-up was not available, so cardiovascular and non-cardiovascular deaths could not be separated. Radiological follow-up for false lumen patency, additional tears and ‘downstream problems’ was not available for much of our historical data.

## 5. Conclusions

AAAD repair remains a procedure with high mortality and morbidity, needing prolonged hospitalization and recuperation. Operative results have improved with changes in operative techniques and cerebral monitoring. Age is not a risk factor for early mortality. Stroke rates and late survival are worse with extended resections. Salvage surgery has poor outcomes. Limited aortic resections have better early and long-term survival.

## 6. Future Directions

Registry data on long-term outcomes remain sparse. Lifelong follow-up is possibly required for registries. This could be achieved by linking to national death registries to capture long-term survival and by including specific causes of death, whether aortic or non-aortic. Data fields to capture the extent of resection and further details contributing to a detailed analysis of perioperative outcomes are also lacking in the databases. These would need to be updated with increasing trends for extended resections, especially with the use of hybrid open stent grafts. These would also inform decisions about re-interventions for delayed complications and progression of disease. There is also a lack of uniformity in data fields and in definitions of datapoints in different national and international aortic databases and registries for inter-registry comparison and meaningful meta-analysis of results. Further research on cerebral perfusion and protection strategies is needed to reduce the high morbidity of adverse neurological outcomes in the perioperative period. Salvage surgery still has very high operative morbidity and mortality for AAAD. Further research on operative techniques and open/percutaneous stent grafts may be needed to improve these results.

## Figures and Tables

**Table 1:** Operative and postoperative outcomes for unmatched and propensity-matched data.

UNMATCHED							
	Overall (*n* = 440)		Limited Resection (*n* = 215)		Extended Resection (*n* = 225)		
	*n*	% or IQR	*n*	% or IQR	*n*	% or IQR	*p* Value
Bypass time	187	(130–249)	173	(121–256)	191	(145.5–245.5)	0.225
Cross-clamp time	99	(75–130)	99	(74–1310	99	(76–128)	0.862
Circulatory arrest time	27	(75–130)	26	(19–33)	27	(18–33.5)	0.545
LOS, days [IQR]	12	(8–20)	12	(8–20)	11	(7–17)	0.012
30-day survival	390	88.6	200	93.0	190	84.4	0.005
Composite outcome	126	28.6	53	24.7	73	32.4	0.071
Stroke/TIA	81	18.4	35	16.3	46	20.4	0.474
In-hospital death	44	10.0	13	6.0	31	13.8	0.007
Overall survival	286	65.0	169	78.6	117	52.0	<0.001
-							
**PROPENSITY-MATCHED**							
	**Overall** **(*n* = 218)**		**Limited Resection** **(*n* = 109)**		**Extended Resection** **(*n* = 109)**		
	***n***	**% or IQR**	***n***	**% or IQR**	***n***	**% or IQR**	***p* Value**
Bypass time	188	(125–249)	172	(119–254)	192	(129–240)	0.828
Cross-clamp time	97	(76–130)	97	(76–130)	97	(78–132)	0.994
Circulatory arrest time	26	(17–35)	26	(17–36)	27	(18–34)	0.848
LOS, days [IQR]	12	(8–18)	12	(9–20)	11	(8–17.5)	0.537
30-day mortality	20	9.2	9	8.3	11	10.1	0.815
Composite outcome	58	26.6	24	22.0	34	31.2	0.133
Stroke/TIA	34	15.6	12	11.0	22	20.2	0.064
In-hospital death	16	7.3	8	7.3	8	7.3	1.000
Overall survival	140	64.2	84	77.1	56	51.4	<0.001
LOS: length of stay, TIA: transient ischemic attack							

**Table 2:** Univariate and multivariate analysis for composite outcome of TIA/stroke/in-hospital death.

	OR	95% CI (Lower, Upper)		*p* Value	OR	95% CI (Lower, Upper)		*p* Value
Extended resection	1.47	0.97	2.23	0.071	1.52	0.92	2.49	0.101
Male gender	1.04	0.67	1.59	0.868				
CCS III-IV	1.37	0.77	2.43	0.282				
NYHA III-IV	1.33	0.75	2.36	0.326				
Prior cardiac surgery	1.28	0.65	2.51	0.48				
Diabetes	1.03	0.42	2.54	0.953				
Hypertension	1.29	0.84	1.98	0.246	1.38	0.83	2.31	0.217
Smoking history	0.96	0.63	1.47	0.859				
Renal disease	1.40	0.46	4.26	0.553				
Pulmonary disease	1.37	0.73	2.58	0.321				
PVD	1.27	0.47	3.46	0.641				
LVEF moderate/poor	1.58	0.94	2.64	0.081	1.49	0.81	2.75	0.203
Hemodynamically unstable	1.47	0.86	2.51	0.159	1.40	0.74	2.65	0.299
Salvage surgery	2.26	1.12	4.55	0.023	2.94	1.22	7.08	0.016
Obese (BMI > 28)	0.84	0.54	1.31	0.451				
Bypass time	1.00	1.00	1.01	<0.001	1.01	1.00	1.01	0.001
Cross-clamp time	1.00	1.00	1.01	0.044	1.00	0.99	1.00	0.724
Circulatory arrest time	1.01	0.99	1.92	0.471				
Additional cardiac procedures	1.19	0.72	1.96	0.51				
Aortic arch procedure	1.41	0.87	2.30	0.167	1.03	0.56	1.91	0.928
Log euroscore	1.03	0.99	1.07	0.163	1.01	0.97	1.06	0.632
Age > 70	1.63	1.07	2.48	0.023	1.85	1.11	3.07	0.017

OR: odds ratio, CI: confidence interval, CCS: Canadian Cardiovascular Score, NYHA: New York Heart Association, PVD: peripheral vascular disease, LVEF: left ventricular ejection fraction, BMI: body mass index.

**Table 3:** Cox proportional hazards analysis for long-term survival.

	HR	95% CI (Lower, Upper)		*p* Value	HR	95% CI (Lower, Upper)		*p* Value
Extended resection	1.90	1.24	2.92	0.003	2.06	1.31	3.25	0.002
Male gender	1.22	0.82	1.81	0.323				
CCS III-IV	1.38	0.80	2.38	0.253				
NYHA III-IV	2.03	1.28	3.22	0.003	2.07	1.28	3.35	0.003
Prior cardiac surgery	1.66	0.99	2.79	0.057	2.01	1.17	3.45	0.012
Diabetes	0.69	0.28	1.69	0.411				
Hypertension	1.59	1.05	2.40	0.027	1.45	0.94	2.22	0.092
Smoking history	1.10	0.75	1.62	0.628				
Renal disease	1.72	0.70	4.23	0.238	1.89	0.75	4.76	0.175
Pulmonary disease	0.84	0.44	1.62	0.613				
PVD	1.13	0.46	2.77	0.796				
LVEF moderate/poor	1.34	0.84	2.12	0.22	1.42	0.88	2.30	0.153
Hemodynamically unstable	0.85	0.48	1.50	0.574				
Salvage surgery	0.79	0.32	1.94	0.602				
Obese (BMI > 28)	0.92	0.61	1.39	0.682				
Bypass time	1.00	0.99	1.00	0.707				
Cross-clamp time	0.99	0.99	1.00	0.136	1.00	0.99	1.00	0.341
Circulatory arrest time	0.99	0.98	1.01	0.343				
Additional cardiac procedures	0.68	0.35	1.31	0.245	0.76	0.37	1.57	0.459
Aortic arch procedure	1.21	0.76	1.93	0.415				
Log euroscore	1.02	0.98	1.06	0.283				
Age > 70	2.10	1.42	3.12	<0.001	2.00	1.32	3.03	0.001

OR: odds ratio, CI: confidence interval, CCS: Canadian Cardiovascular Score, NYHA: New York Heart Association, PVD: peripheral vascular disease, LVEF: left ventricular ejection fraction, BMI: body mass index.

**Table 4:** Re-operations after acute aortic dissection repair.

Aortic Procedures after AAAD Repair		
Aortic valve and root		(47.2%)
Aortic root repair/replacement	10	
Aortic valve replacement +/− redo interposition graft	7	
		
**Arch**		(13.9%)
Arch resection +/− frozen elephant trunk	5	
		
**Descending thoracic aorta**		(27.8%)
Descending thoracic aortic aneurysm repair	8	
Thoracic endovascular aneurysm repair	2	
		
**Abdominal aortic aneurysm repair**		(8.3%)
Abdominal aortic aneurysm repair (open)	2	
Endovascular aneurysm repair (EVAR)	1	
Total	36	(100%)

## Data Availability

The data presented in this study are available on request from the corresponding author due to institutional data protection policies.

## Supplementary Materials

The following supporting information can be downloaded at: https://www.mdpi.com/article/10.3390/medicina60081245/s1, Supplementary Figure S1. Standardized bias across covariates in propensity match model; Supplementary Figure S2. Survival by type of neuroprotection A. Unmatched, B. Matched; Supplementary Figure S3. Survival by age. Unmatched (A) and propensity-matched (B) cohorts. Supplementary Table S1. Demographic characteristics (unmatched cohort); Supplementary Table S2. Propensity matched demographics; Supplementary Table S3. Univariate and multivariate analysis for in hospital death; Supplementary Table S4. Long term survival in unmatched and propensity matched data.
