# Supplementary material for: Limited vs. Extended Resection of Stanford Type A Acute Aortic Dissections
Source: Medicina (Kaunas). 2024 Jul 31;60(8):1245. doi: 10.3390/medicina60081245 (PMC11356285; doi:10.3390/medicina60081245)

Appendix/ Supplementary data

Supplementary Table S1. Demographic characteristics (unmatched cohort)

	**Overall (n=440)**		**Limited Resection (n=215)**		**Extended Resection (n=225)**		**P value**
	**N**	**%**	**N**	**%**	**n**	**%**	
Euroscore [IQR]	9.5	[7-12]	9	[7-12]	10	[7-12]	0.918
Age [IQR]	66.7	[56-74]	66	[55.1-73.6]	67.8	[57.3-74.4]	0.264
Male	282	64.1	137	63.7	145	64.4	0.874
NYHA III-IV	62	14.1	28	13.0	34	15.1	0.529
CCS III-IV	61	13.9	29	13.5	32	14.2	0.824
Prior cardiac surgery	42	9.5	22	10.2	20	8.9	0.632
Diabetes	24	5.5	10	4.7	14	6.2	0.468
Hypertension	264	60.0	123	57.2	141	62.7	0.243
Smoker	181	41.1	83	38.6	98	43.6	0.569
Renal disease	14	3.2	4	1.9	10	4.4	0.123
Pulmonary disease	49	11.1	24	11.2	25	11.1	0.986
Obese (BMI >28)	148	33.6	65	30.2	83	36.9	0.44
PVD	18	4.1	11	5.1	7	3.1	0.47
LVEF <30%	79	18.0	39	18.1	40	17.8	0.921
Salvage surgery	35	8.0	18	8.4	17	7.6	0.752
Aortic arch procedure	93	21.1	45	20.9	48	21.3	0.918
Additional cardiac procedure	89	20.2	54	25.1	35	15.6	0.013
Unstable	74	16.8	39	18.1	35	15.6	0.75

IQR: interquantile range, BSA: body surface area, CCT: cross clamp time, CBP: cardiopulmonary bypass, CAT: circulatory arrest time, NYHA: New York Heart Association, CCS: Canadian Cardiovascular Society, TIA: transient ischemic attack, CVA: cerebrovascular accident, PVD: peripheral vascular disease, LVEF: left ventricular ejection fraction.

Supplementary Table S2. Propensity matched demographics

	Limited Resection (n=109)		Extended Resection (n=109)		
	N	%	N	%	P value
Age >75	21	19.3	20	18.3	1.000
Male	68	62.4	71	65.1	0.775
NYHA III-IV	9	8.3	10	9.2	1.000
CCS III-IV	18	16.5	10	9.2	0.169
Prior cardiac surgery	7	6.4	6	5.5	1.000
Diabetes	2	1.8	2	1.8	1.000
Hypertension	62	56.9	63	57.8	1.000
Smoker	46	42.2	44	40.4	0.892
Renal disease	0	0.0	0	0.0	1.000
Pulmonary disease	4	3.7	5	4.6	1.000
Obese (BMI >28)	4	3.7	5	4.6	1.000
PVD	9	8.3	9	8.3	1.000
LVEF <30%	11	10.1	11	10.1	1.000
Salvage surgery	3	2.8	3	2.8	1.000
Additional cardiac procedure	9	8.3	10	9.2	1.000
Unstable	22	20.2	25	22.9	0.743

IQR: interquantile range, BSA: body surface area, CCT: cross clamp time, CBP: cardiopulmonary bypass, CAT: circulatory arrest time, NYHA: New York Heart Association, CCS: Canadian Cardiovascular Society, TIA: transient ischemic attack, CVA: cerebrovascular accident, PVD: peripheral vascular disease, LVEF: left ventricular ejection fraction.

Supplementary Table S3. Univariate and multivariate analysis for in hospital death

	OR	95% CI		P value	OR	95% CI		P value
		lower	Upper			lower	upper	
Extended Resection	2.48	1.26	4.89	0.008	2.92	1.36	6.29	0.006
Male gender	0.64	0.32	1.29	0.211	0.57	0.26	1.27	0.171
CCS III-IV	2.31	1.10	4.85	0.028	1.93	0.83	4.49	0.124
NYHA III-IV	3.38	1.68	6.83	0.001	2.49	1.10	5.63	0.029
Prior cardiac surgery	1.58	0.63	3.99	0.334				
Diabetes	1.31	0.37	4.57	0.675				
Hypertension	1.89	0.94	3.77	0.073	1.70	0.79	3.67	0.176
Smoking history	1.65	0.88	3.08	0.116	1.51	0.73	3.11	0.267
Renal disease	2.56	0.69	9.55	0.162	1.31	0.28	6.08	0.734
Pulmonary disease	0.78	0.27	2.28	0.65				
PVD	1.87	0.52	6.74	0.338				
LVEF moderate/poor	1.84	0.90	3.76	0.094	1.29	0.56	2.99	0.554
Hemodynamically unstable	2.24	1.10	4.56	0.026	1.66	0.70	3.92	0.252
Salvage surgery	3.66	1.59	8.42	0.002	3.60	1.27	10.17	0.016
Obese (BMI >28)	0.72	0.36	1.44	0.348				
Additional cardiac procedures	2.00	1.01	3.95	0.047	2.23	1.01	4.92	0.047
Aortic arch procedure	1.28	0.62	2.63	0.509				
Log euroscore	0.96	0.84	1.09	0.495				
Age >70	1.58	0.85	2.96	0.152	1.86	0.90	3.84	0.094

OR: odds ratio, CI: confidence interval, CCS: Canadian cardiovascular score, NYHA: New York Heart Association, PVD: peripheral vascular disease, LVEF: left ventricular ejection fraction, BMI: body mass index

Supplementary Table S4. Long term survival in unmatched and propensity matched data.

	UNMATCHED									
	Overall (n=440)			Limited Resection (n=215)			Extended Resection (n=225)			
	%	n at risk	deaths	%	n at risk	deaths	%	n at risk	deaths	P value
**Unmatched**										
1-year	83.4	440	71	87.6	215	26	79.4	225	45	0.929
3-year	77.4	346	23	85.5	179	4	69.5	167	19	0.015
5-year	73.4	262	12	83.4	142	3	64.1	120	9	0.003
10-year	58.6	204	32	69	105	12	49.6	99	20	0.008
15-year	44.1	80	14	64.3	22	1	34.2	58	13	0.004

	PROPENSITY MATCHED									
	Overall (n=218)			Limited Resection (n=109)			Extended Resection (n=109)			
	%	n at risk	deaths	%	n at risk	deaths	%	n at risk	deaths	P value
**Propensity-matched**										
1-year	87.6	218	27	88.07	109	13	87.16	109	14	0.727
3-year	79.3	191	17	85.19	96	3	73.29	95	14	0.029
5-year	75.2	151	7	82.87	84	2	67.61	67	5	0.01
10-year	62.1	118	16	68.37	61	7	55.79	57	9	0.06
15-year	46.8	49	9	68.37	12	0	38.17	37	9	0.024

Supplementary Figure legends

Supplementary Figure S1. Standardized bias across covariates in propensity match model

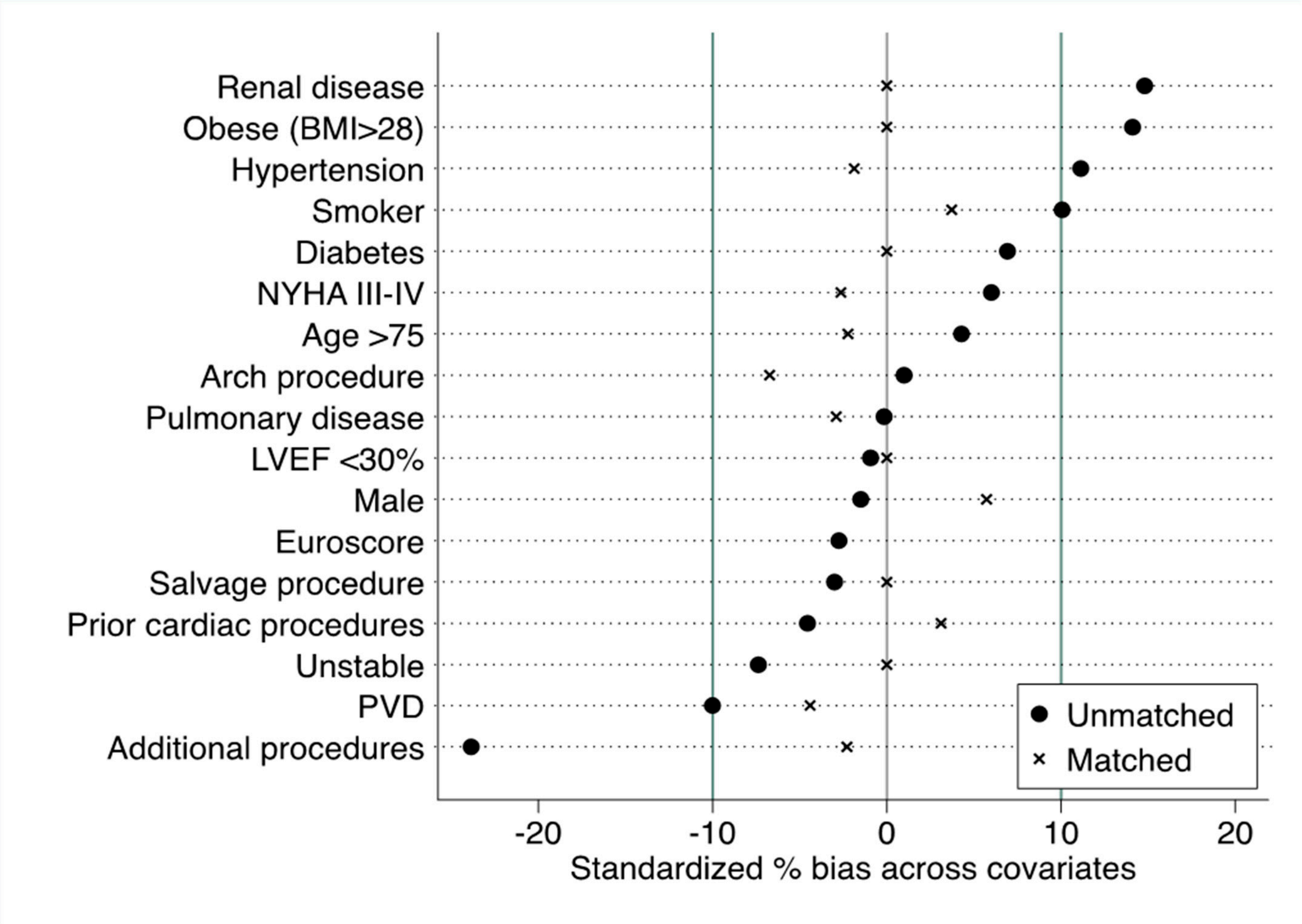

Supplementary Figure S2. Survival by type of neuroprotection A. Unmatched, B. Matched

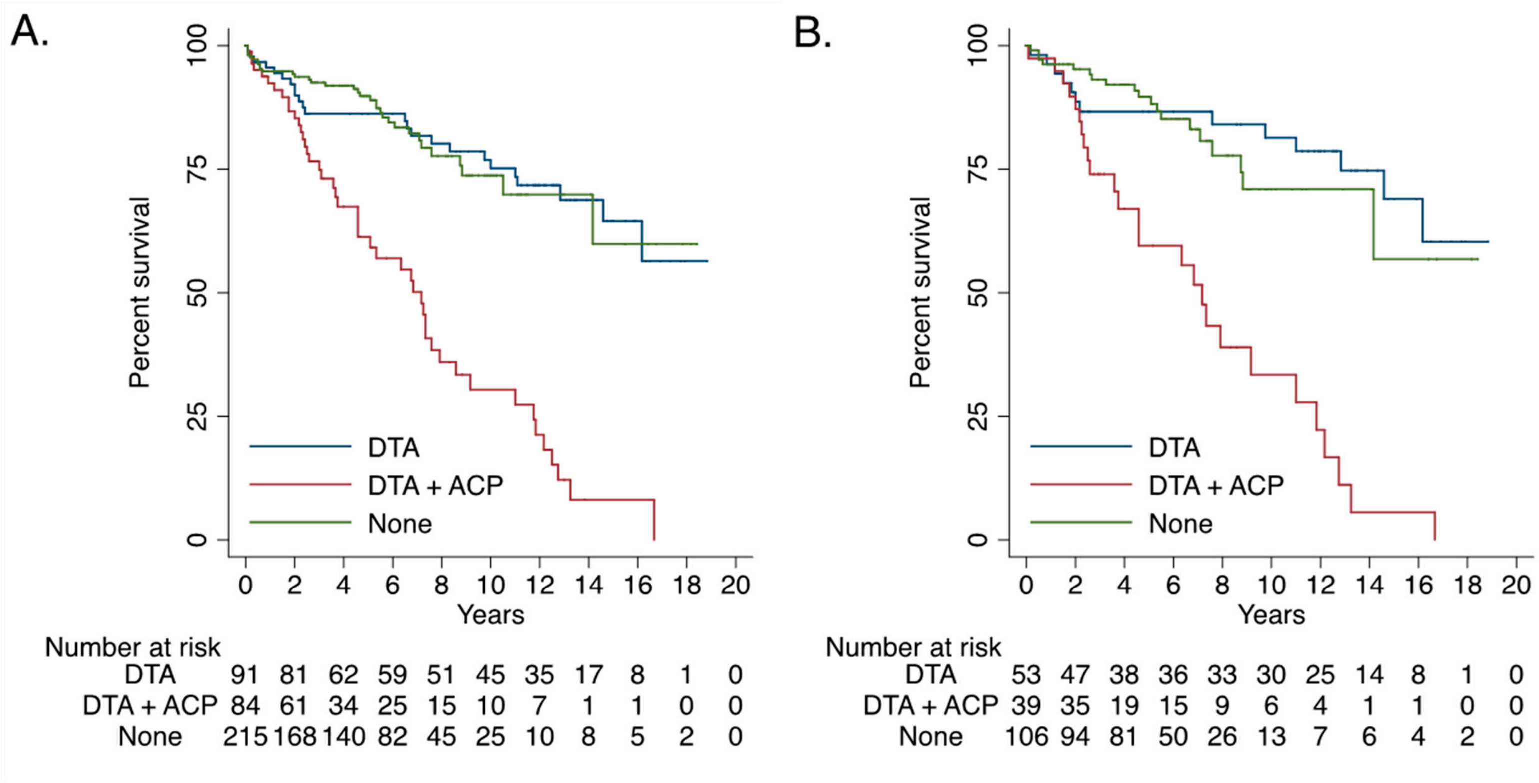

Supplementary Figure S3. Survival by age. Unmatched (A) and propensity-matched (B) cohorts

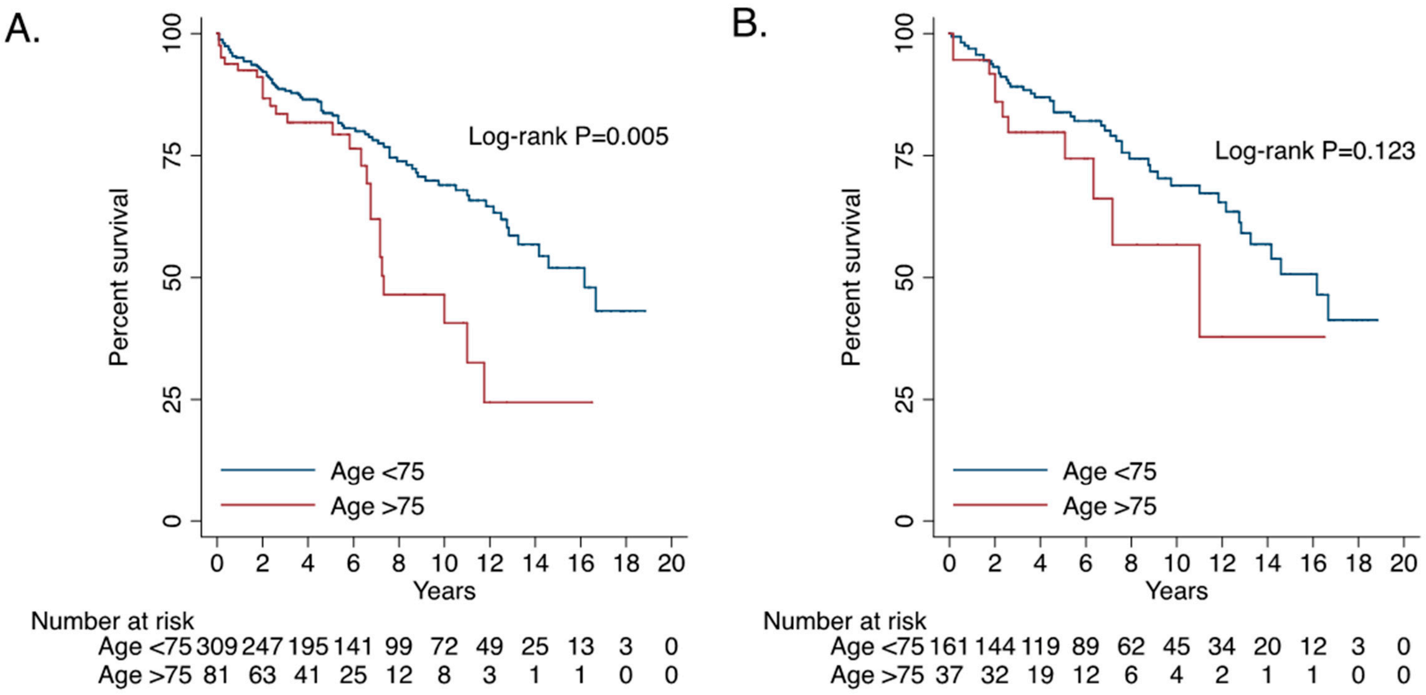

Supplement: Supplementary file 1 [file medicina-60-01245-s001.zip › medicina-3102214-supplementary.pdf]
